# Host Responses to Sepsis Vary in Different Low-Lethality Murine Models

**DOI:** 10.1371/journal.pone.0094404

**Published:** 2014-05-01

**Authors:** Lori F. Gentile, Dina C. Nacionales, M. Cecilia Lopez, Erin Vanzant, Angela Cuenca, Benjamin E. Szpila, Alex G. Cuenca, Anna Joseph, Frederick A. Moore, Christiaan Leeuwenburgh, Henry V. Baker, Lyle L. Moldawer, Philip A. Efron

**Affiliations:** 1 Departments of Surgery, University of Florida College of Medicine, Gainesville, Florida, United States of America; 2 Molecular Genetics and Microbiology, University of Florida College of Medicine, Gainesville, Florida, United States of America; 3 Institute on Aging, University of Florida College of Medicine, Gainesville, Florida, United States of America; University of Cincinnati, United States of America

## Abstract

**Introduction:**

Animal models for the study of sepsis are being increasingly scrutinized, despite their essential role for early translational research. In particular, recent studies have suggested that at the level of the leukocyte transcriptome, murine models of burns, trauma and endotoxemia markedly differ from their human equivalents, and are only weakly similar amongst themselves. We compared the plasma cytokine and leukocyte transcriptome responses between two different low-lethality murine models of polymicrobial intra-abdominal sepsis.

**Methods:**

Six to ten week male C57BL/6j mice underwent either the ‘gold standard’ cecal ligation and puncture (CLP) model of intra-abdominal sepsis or administration of a cecal slurry (CS), where cecal contents are injected intraperitoneally. Surviving mice were euthanized at two hours, one or three days after sepsis.

**Results:**

The murine leukocyte transcriptomic response to the CLP and CS models of sepsis was surprisingly dissimilar at two hours, one, and three days after sepsis. The Pearson correlation coefficient for the maximum change in expression for the entire leukocyte transcriptome that changed significantly over time (n = 19,071) was R = 0.54 (R^2^ = 0.297). The CS model resulted in greater magnitude of early inflammatory gene expression changes in response to sepsis with associated increased production of inflammatory chemokines and cytokines. Two hours after sepsis, CLP had more significant expression of genes associated with IL-10 signaling pathways, whereas CS had greater expression of genes related to CD28, apoptosis, IL-1 and T-cell receptor signaling. By three days, the changes in gene expression in both sepsis models were returning to baseline in surviving animals.

**Conclusion:**

These analyses reveal that the murine blood leukocyte response to sepsis is highly dependent on which model of intra-abdominal sepsis is employed, despite their similar lethality. It may be difficult to extrapolate findings from one murine model to another, let alone to human sepsis.

## Introduction

Despite improvements in the diagnosis, treatment and management of sepsis and septic shock over the last several decades, sepsis continues to represent a significant cause of morbidity and mortality across all age ranges worldwide [1], [2]. Mortality from sepsis alone is reported to range from 28–50%, and death is more frequent in the pediatric and elderly populations [3], [4]. Even with recent improvements in outcomes due to changes in practice, the incidence and mortality from sepsis is increasing, particularly in the elderly population, and sepsis continues to remain the leading cause of ICU mortality, prolonged ICU stays and multiple organ failure (MOF)[3]–[5].

It has long been known that animal models do not fully recapitulate the human condition; however, considering the numerous recent failures of clinical trials based on positive outcomes in animal studies [1], [6]–[8], recent criticisms of animal models of sepsis and injury have blossomed [9]. A recent controversial report has revealed that at the level of the blood leukocyte transcriptome, the human response to trauma, burns and endotoxicosis is remarkably similar, whereas the comparison of the human response to murine models of injury was surprisingly poor (9). More interestingly, the murine transcriptomic responses to burn, trauma and endotoxicosis exhibited very little similarity among themselves.

As animal models of sepsis will continue to remain essential for early translational research, understanding the limitations of these models is essential [9]. Additionally, investigators must take into consideration the precise human condition they are studying and strive to use a murine model that best recapitulates the human responses being studied [10]. Oftentimes, murine models may only model a single component of the human response to severe sepsis or the systemic inflammatory response syndrome (SIRS). For instance, highly lethal models of cecal ligation and puncture (CLP), which are considered the ‘*gold* standard’ [11] animal model of intra-abdominal polymicrobial sepsis, appear to emphasize an early exaggerated inflammatory response, whereas, reduced lethality models, tend to emphasize a requirement for antimicrobial responses [12], [13].

In this report we examine two commonly used murine models of polymicrobial, intra-abdominal sepsis. Both models mimic the low mortality seen in human severe sepsis, but the source of sepsis is somewhat different, as one arises from a cecal nidus of infection (CLP) and the other from the bolus administration of cecal contents (CS). We sought to examine similarities and differences in the model at the level of both the plasma cytokine responses and the blood leukocyte transcriptome. Surprisingly, we find that changes in the murine leukocyte transcriptome to these relatively similar models of abdominal sepsis are more dissimilar to each other than the reported differences in gene expression between humans with burns and trauma. Interestingly, signaling pathways activated by CLP and CS are also fundamentally different, with the former emphasizing down regulation of T cell activation pathways, and the latter emphasizing the early inflammatory response.

## Materials and Methods

### Mice

Male C57BL/6J mice, age 6–10 weeks, were purchased from Jackson Laboratory (Bar Harbor, ME, USA) and were used in experiments approved by the University of Florida IACUC (approval number: 201106451). Mice were housed in pathogen-free facilities and acclimated for at least one week prior to use.

### Cecal Slurry

Cecal contents were harvested from adult C57BL/6J mice, weighed, and suspended in 5% dextrose to make a cecal content slurry at a concentration of 80 mg/ml as previously published [14]. This was then injected intraperitoneally at a dose of 1.3 mg/gm body weight for a goal of an estimated 30% lethal dose (LD_30_).

### Cecal Ligation and Puncture

CLP was performed using isoflurane anesthesia as previously described [15]. A small one centimeter laparotomy was performed; the cecum was exposed and then ligated using a 2-0 silk suture. After ligation, the cecum was punctured through both walls using a 25-gauge needle. The cecum was then returned to the abdomen and the incision was closed using surgical staples. After the surgical procedure, mice were placed on a warming blanket where they were administered 0.05–0.2 mg/kg of buprenorphine every 12 hours for 24 hours, returned to their cage, and monitored for signs of distress.

Surviving mice were euthanized at two hours, one and three days following sepsis, and whole blood was collected via intra-cardiac puncture, used for complete blood count (CBC) with differential determination, RNA isolation, or determination of plasma cytokine response. An additional group of animals were followed for seven days to judge long-term survival. All animals were monitored every 6–8 hours for signs of distress and endpoints including hunching, decreased socialization, anorexia, weight loss of 15% or more, the inability to evade handling and the inability to right themselves when placed on their side for adult animals. Neonatal mice were monitored for signs of distress, neglect by mother, or signs of poor feeding/dehydration (absence of milk in the stomach which can be seen externally). Animals meeting these criteria were humanely euthanized via CO_2_ asphyxiation followed by decapitation for neonates and cervical dislocation for adults, and were considered non-survivors.

### Gene Expression Profiling and Microarray Analysis

Whole blood was collected via intra-cardiac puncture at two hours, one and three days after the onset of sepsis, using one milliliter syringes containing 100 µl of 169 mM EDTA. Red blood cells were lysed using Buffer EL (Qiagen, Valencia, CA), and the supernatant was decanted after centrifugation. The cell pellet was homogenized in RLT buffer (Qiagen, Valencia, CA) supplemented with 2-mercaptoethanol and passed through QiaShredder™ (Qiagen, Valencia, CA). Total RNA was isolated using RNeasy™ kit (Qiagen, Valencia, CA), and the quality and quantity were assessed using an Agilent Bioanalyzer 2000. Nucleic acids were labeled using the 3′ IVT Express Kit and 15 µg of labeled cRNA was hybridized to Mouse Genome 430 2.0 Arrays (Affymetrix, Santa Clara, CA). Arrays were hybridized for 16 hours at 45°C. Following hybridization, arrays were stained and washed using an FS450 Affymetrix fluidics station and Affymetrix FlexFS 450-0004 protocol. Arrays were then scanned in an Affymetrix GeneChip™ scanner 7G Plus. The gene expression data were submitted to the Gene Expression Omnibus (GEO) database with the accession number GSE55238.

### Plasma Cytokines

Plasma cytokine concentrations were determined using the multiplex Luminex™ platform on 50 µl of plasma. Samples were run in duplicate.

### Statistical Methods

A log2 transformed expression matrix was calculated using RMA™ as implemented in the Partek Genomic Suite 6.6 (Affymetrix, Santa Clara, CA). Expression patterns were compared between healthy control, young adult mice and septic mice, and sepsis responsive genes were considered significant with a p-value of p<0.001 (F-test). Leave-one out cross validation was performed to compute the misclassification rate, and Monte Carlo simulation was used to determine if the miscalculation rate was significantly better than predicted by chance. Once significant genes were identified, fold changes were calculated from the genomic response between septic and healthy control mice. Pearson linear correlations were calculated on the changes in log2 transformed expression data to assess the correlation between changes in the CLP and CS models of murine sepsis over time.

### Pathway Analysis

Functional pathway analysis was performed using Ingenuity Pathway Analysis (IPA, Redwood City, CA, USA), which allows for the discovery of signaling pathways associated with the dataset of interest [16]. Only genes that changed significantly with a p<0.001 and had greater than two-fold change from control mice for each model were subjected to functional analysis. IPA performs a functional pathways analysis as part of its tools available to researchers, in which they identify those pathways that are over-represented, indicating that their expression is affected by the intervention. Significance was determined using a Z-score. Values of 2< Z<−2 are considered significant and correspond to a 95% confidence interval. Additionally, the IPA canonical pathway analysis displays pathways that are most associated with the genes in our dataset and significance is calculated at a p<0.05 level of significance. Within the canonical pathway analysis, the Molecule Activity Predictor tool was used to determine whether the overall effects on the pathway were activated or inhibited.

## Results

### The Murine Leukocyte Transcriptomic Response to the CLP and CS Models of Sepsis Are Genomically Dissimilar Two Hours, One Day and Three Days after the Onset of Sepsis

Although many models of murine sepsis exist, CLP remains the gold-standard model of intra-abdominal sepsis in mice [11]. The CS model of sepsis is similar in scope to the newer colon ascendans peritoneal stent (CASP) model of intra-abdominal sepsis which is thought to more closely mimic a human generalized peritonitis response, in that it represents acute generalized peritonitis. It is particularly helpful in those mice where a CLP is not feasible, such as neonatal mice [14]. Both models are thought to induce an immune response closely mimicking human intra-abdominal sepsis. In order to compare the two models, we isolated RNA from total leukocytes at two hours, one and three days following a low-lethality (LD_20–30_) model of either CS induced sepsis or CLP induced sepsis and genome-wide expression analysis was performed.

**Figure 1:**
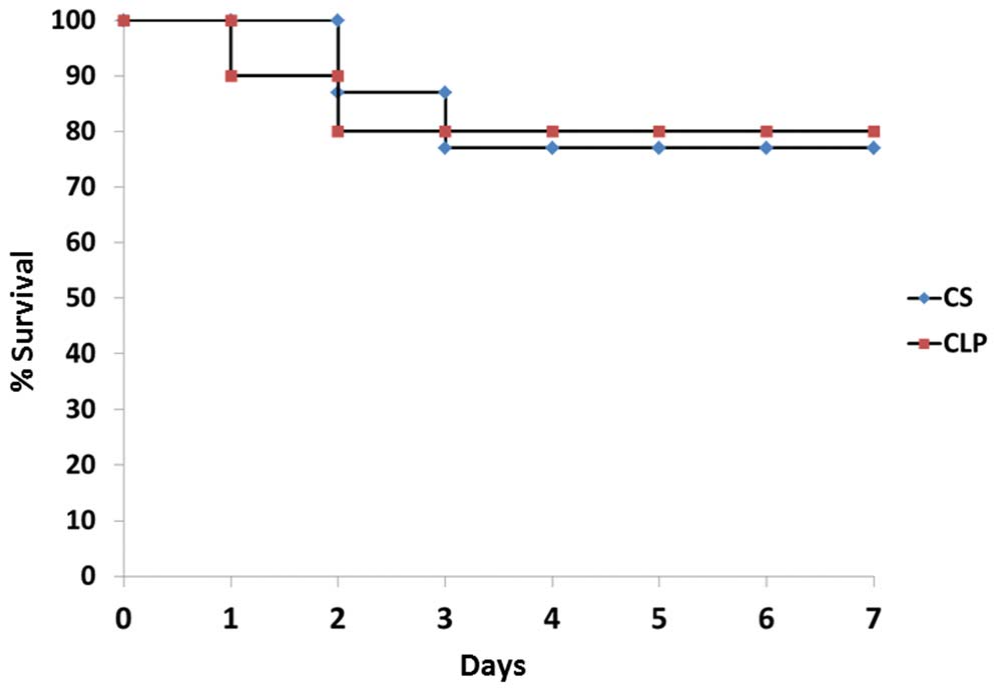
Survival responses to a cecal ligation and puncture (CLP) versus a cecal slurry (CS) model of polymicrobial sepsis. Survival was evaluated out to seven days. Mortality in the two models was similar. In the CS model, mice died on the second and third days, whereas, in the CLP model mice died on days one and two. (•- CS, ▪ - CLP).

As shown in Figure 1, mortality in the two models was similar although the kinetics of mortality may be slightly different. In low-lethality sepsis models of CLP and CS it appears that mice tend to die early (days 1–3) and there is no statistical difference. In the CS model, mice died on the second and third days, whereas, in the CLP model mice died on days one and two.

**Figure 2:**
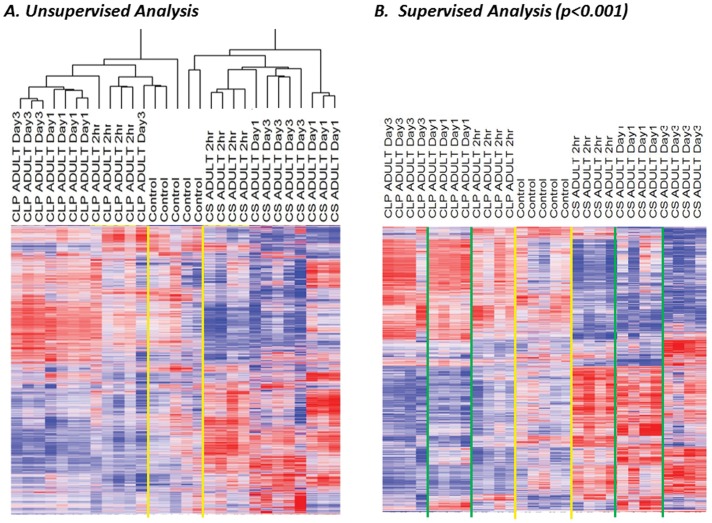
The CLP and CS models of murine intra-abdominal sepsis each induce a distinct genomic response after sepsis. **A**. Unsupervised cluster analysis with a coefficient of variation of >0.5 reveals that the expression of 19,071 probe sets (12,838 genes) varied after sepsis, and segregated based on the type of sepsis model employed. **B**. A supervised analysis shows that there were 11,612 probesets (7,581 genes) differentially expressed after sepsis (p<0.001) and the expression patterns from these two models appear distinct from one another.

Using a simple unsupervised analysis, we first asked whether the two models of sepsis altered the gene expression of blood leukocytes at either two hours, one day or three days. Using cluster analysis and an individual probe set coefficient of variance threshold of greater than 0.5, we found that there were 19,071 probe sets (representing 12,838 genes) whose expression varied. Surprisingly, when these genes were clustered, the main node of separation was the sepsis model employed rather than timing of sample (Figure 2A). Using a supervised analysis, and setting the threshold at p<0.001 by F test, there were 11,612 probe sets (representing 7,581 genes) that were significantly altered after sepsis, and by examining the heat map (Figure 2B), one can see that the genomic response induced by the CS model of intra-abdominal sepsis appears distinct from that induced by the CLP model of sepsis. These differences in gene expression between the three classes (CLP, CS and healthy control) could be used to identify the source of the sample (p<0.01), as confirmed by leave-one out cross validation analysis with Monte Carlo simulation.

**Figure 3:**
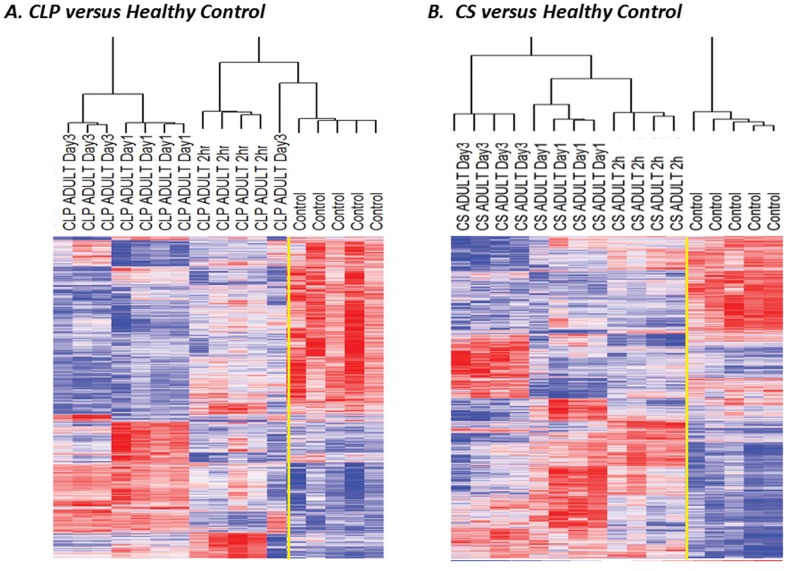
Heat maps from septic mice two hours, one day, and three days after sepsis reveal that CS induces a leukocyte transcriptomic response that is distinct from CLP. After CLP there were 2,869 probe sets, representing 2,159 genes that were differentially expressed between septic and healthy control mice that were significant at p<0.001 across all time points. After CS there were 4,486 probe sets, representing 3,305 genes that were differentially expressed. There were only 802 probe sets (representing 757 genes) that were the same amongst the two models.

Further analysis was subsequently performed by examining the changes in gene expression from mice undergoing sepsis from each model compared to healthy control mice. We found that after CLP there were 2,869 probe sets, representing 2,159 genes, that were differentially expressed between septic and healthy control mice (p<0.001). After CS, there were 4,486 probe sets, representing 3,305 genes, that were differentially expressed (as compared to healthy control mice (p<0.001) (Figure 3). In depth analysis revealved that only 757 of the genes that were significantly altered following sepsis overlapped, and changed in both models. Instead, the majority of genes, 1,314 and 2,460, were unique to both CLP and CS, respectively.

**Figure 4:**
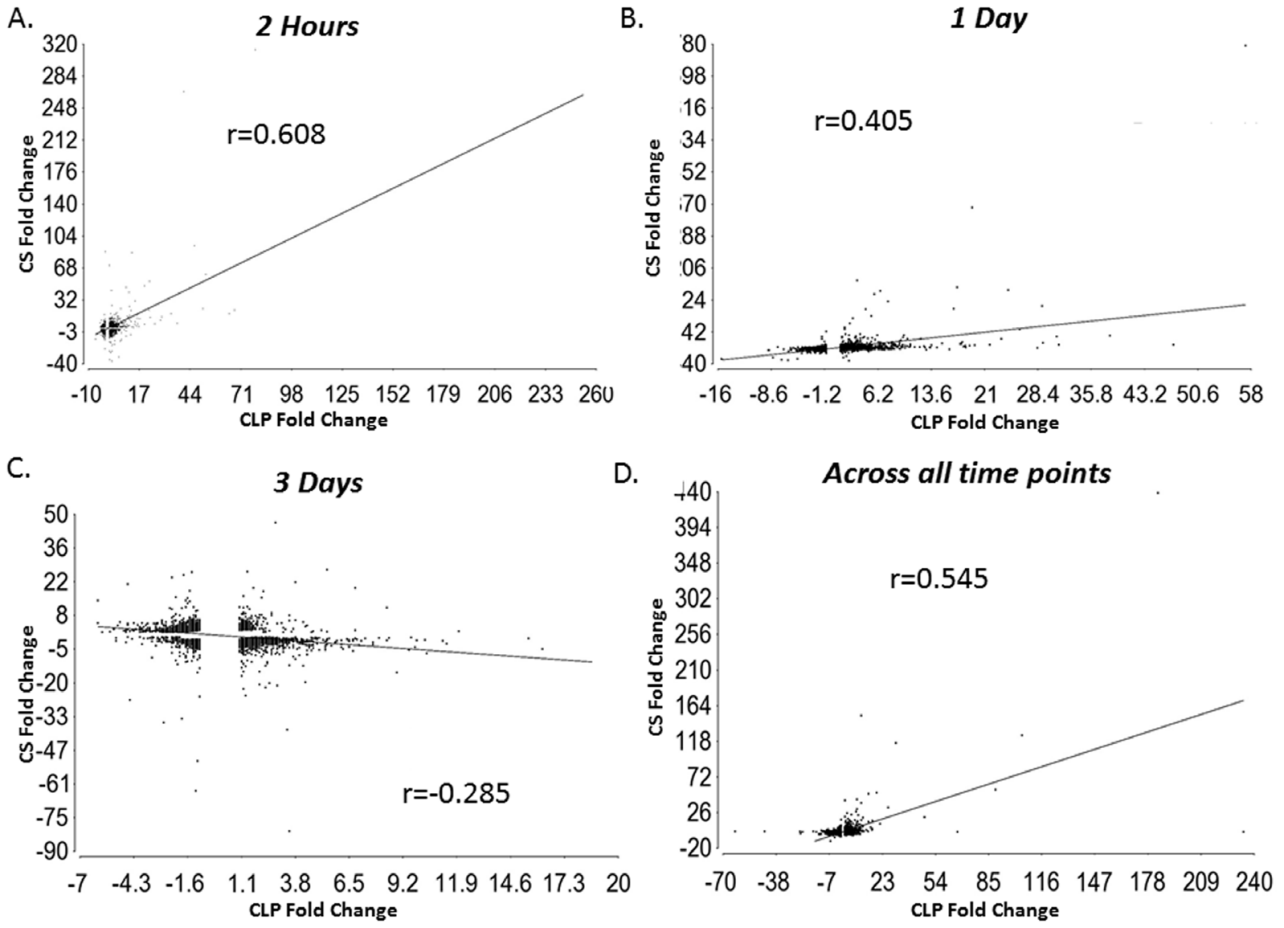
The CLP and CS models of intra-abdominal sepsis have the highest genome-wide linear correlation early after sepsis. Two hours, one day and three days after sepsis genome-wide linear correlations examining the mean change in expression for all sepsis responsive genes significant at a p-value of p<0.001 between the CLP and CS models were performed. **A**. The models were most similar to each other at the two hour time point with an r = 0.608. **B**. They were less similar one day after sepsis with an r = 0.405. **C**. Three days following sepsis the two models had a negative correlation with an r = −0.284. **D**. The genome-wide linear correlations of the maximum change in gene expression for each model over time was r = 0.545.

We also performed genome-wide linear correlations where we examined the mean change in expression for all sepsis responsive genes significant at a p-value of p<0.001 between the CLP and CS models at two hours, one day and three days after sepsis (Figure 4). The models were most similar to each other at the two hour time point with a Pearson correlation coefficient r = 0.608 (p<0.0001) (Figure 4A). They were less similar one day after sepsis with an r = 0.405 (p<0.0001) (Figure 4B). Three days following sepsis the two models had an inverse correlation that was highly significant (p<0.0001) with an r = −0.284 (Figure 4C). We also examined the the maximum change in gene expression for each model over time and found the Pearson correlation coefficient to be r = 0.545 (r^2^ = 0.297) (Figure 4D).

### In Both the CLP and CS Models there are Unique Gene Expression Changes and Activated Pathways after Sepsis

Both the CLP and CS models of intra-abdominal sepsis incite an inflammatory response, including the expression of interleukins/chemokines/cytokines and toll-like receptors (TLR). However, there are certain up regulated genes that are unique to each model. For example, *Il-1B*, *Tlr2*, *Tlr3*, *Cxcl10*, *Hmgb2* are up regulated exclusively in the CLP model (Table S1), whereas *Arg1*, *Cd40*, *Cxcr3*, and *Tlr7* are exclusively up regulated in the CS model (Table S2). Additionally, the CS model appears to have greater down regulation of the *MhcII* class of genes. Common immune related genes whose expression is significantly changed in both models include multiple chemokines and cytokines, including, but not limited to, *Il-10*, *Il-6*, *Mip-1α*, and *Tlr4* (Table S3).

### Canonical Pathway Analysis Reveals that CS has Increased Activation of Pathways Involved with the Innate Inflammatory Response, whereas CLP has Greater Inhibition of Pathways Involved in the Adaptive Immune Response

**Figure 5:**
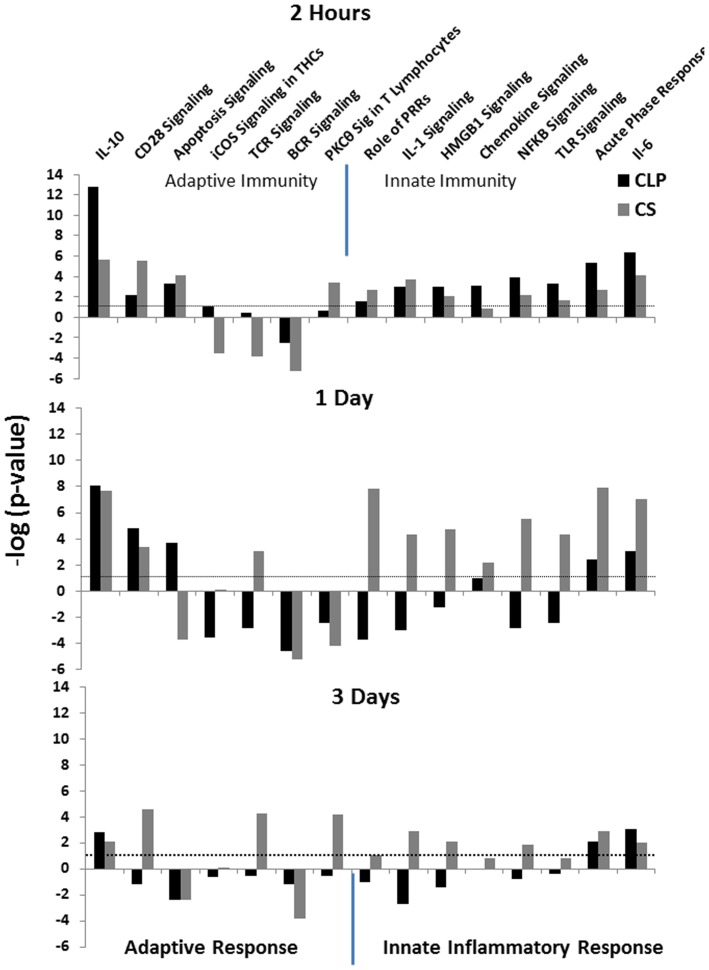
The differential gene expression between CLP and CS leads to the dissimilar activation of various immune related pathways after sepsis. Selected inflammation and immune related signaling pathways from IPA are presented. The –log (p-value) presented on the y-axis represents a measure of how likely the pathway is to contain genes associated with our dataset. (The –log for a p-value of 0.05 is 1.3). Negative values represent those pathways whose actions were inhibited using the Molecule Activity Predictor tool in IPA canonical pathway analysis, and positive values represent those pathways that are activated.

When examining the inflammation and immune related signaling pathways in IPA, we found that the *Il-10* signaling pathway was more significantly expressed after CLP than CS across all three time points and had the greatest proportion of up-regulated genes two hours after sepsis (Figure 5). Additionally, two hours after sepsis, the CLP model had the greatest gene expression related to NFκB signaling, TLR signaling, acute phase response and Il-6 signaling, whereas one and three days after sepsis, the the it actually showed inhibition of many of these inflammatory pathways (Figure 5). The CS model had the greatest gene expression related to innate inflammatory pathways one day after sepsis with activation of pathways related to the role of pattern recognition receptors (PRRs), Il-1, Il-6, Nfkb, and Hmgb1 signaling, chemokine signing, TLR signaling, and the acute phase response (Figure 5). Three days after sepsis both groups of surviving mice had decreased levels of significance in the majority of immune and inflammation related signaling pathways compared to two hours and one day after sepsis and the changes in gene expression from all pathways were returning to baseline in both models. Overall, both models showed up regulation of innate immune inflammatory pathways and down regulation of adaptive immune pathways, and this was most pronounced one day after sepsis (Figure 5).

### In General, as Compared to CLP, the CS Model Results in a Greater Magnitude of Early Inflammatory Gene Expression Changes, with an Associated Increased Production of Inflammatory Chemokines and Cytokines

**Figure 6:**
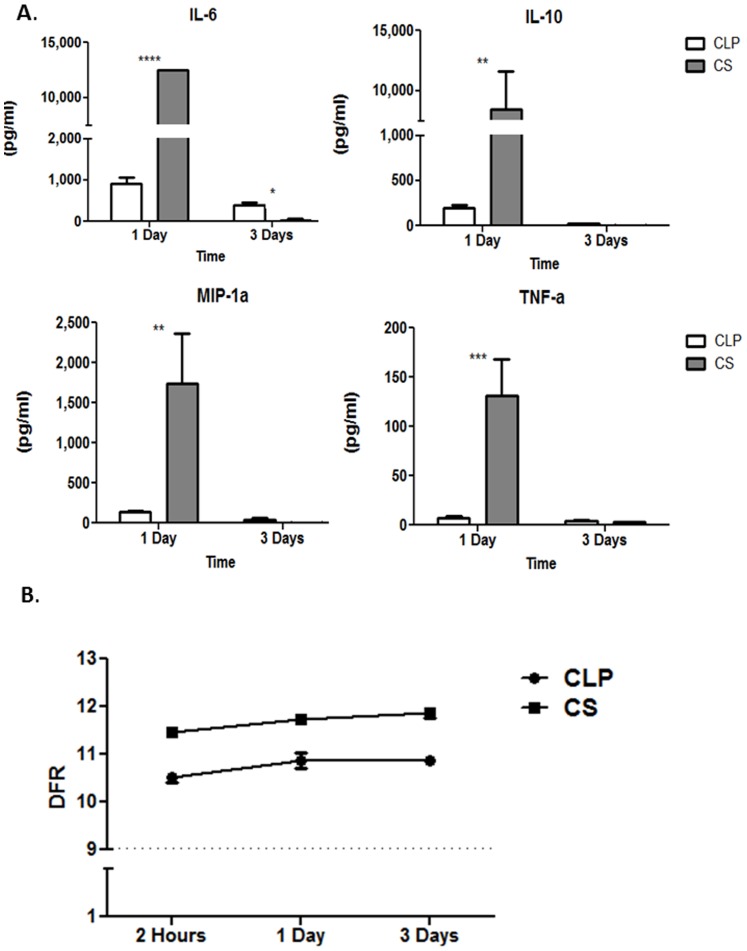
The CS model of intra-abdominal sepsis has a greater magnitude of inflammatory cytokine production than the CLP model 24 hours after sepsis and induces greater gene expression changes than the CLP model. **A**. The CS model had significantly increased production of IL-6, IL-10, MIP1α, and TNFα in the plasma compared to mice who underwent the CLP model of sepsis (p<0.0001, p<0.01, p<0.01, p<0.001 respectively). **B**. A DFR score was calculated to examine the normalized differences in expression for each of the genes from naïve controls and graphed for each model over time. The mean DFR with standard error of the means are graphed on the y-axis and time on the x-axis. All points are significant on 2-way ANOVA (*p<0.0001). The dashed line represents the mean value of naïve controls.

When comparing the fold changes of important immune related genes that are significantly altered after sepsis in both murine models of sepsis, we found that the CS model tends to induce a greater magnitude fold change from baseline than the CLP model (Table S3) for genes involved in inflammation. For example, *Il-6* is up-regulated 31.1, 174.8 and 7.1 fold from control mice at two hours, one day, and three days, respectively, after the onset of CS sepsis. However, in the CLP model, it is up regulated only 14.8, 3.3 and 2 fold from control animals, respectively, at those same time points. These findings are also similar to those found with *Il-10*, *Tnf*, and *Mip-1α*. In addition, when we determined the plasma cytokine levels of these four proteins, we found that at one day after sepsis, the mice undergoing the CS model had significantly increased production of IL-6, IL-10, MIP1α, and TNFα in the plasma compared to mice who underwent the CLP model of sepsis (all p<0.01) (Figure 6A), correlating to their genomic expression.

Finally, we examined the global gene expression changes of the murine response to polymicrobial sepsis in the models over time by examining the ‘distance from reference’ score (DFR) [17]. We have found that heat maps, principle component analysis and individual gene lists make it difficult to globally assess the overall aberrations in gene expression produced by an inflammatory event. We created the DFR to address this problem [18]. The DFR calculates the sum of the normalized differences in expression for each of the significant genes from the mean expression obtained from naïve controls using the equation, 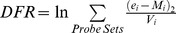 where e_i_ is the gene expression level and M_i_ and V_i_ are the control group mean and variance for the i^th^ probe set [17]. This, in essence, calculates the distance from each subject’s gene expression profile and the profile obtained from healthy control animals. The expression data from significant genes (p<0.001) in each model over time can be reduced to a *single natural log* metric used to evaluate the difference in expression from naïve control mice. We found that the CS model of sepsis produced greater deviation in gene expression from naïve animals at each time point compared to CLP (Figure 6B). At two hours following sepsis, the CS model induced three times the amount of change compared to the CLP model with 2242 genes that are significantly altered from baseline compared to only 673 genes that are altered in response to sepsis after the CLP model was performed.

**Figure 7:**
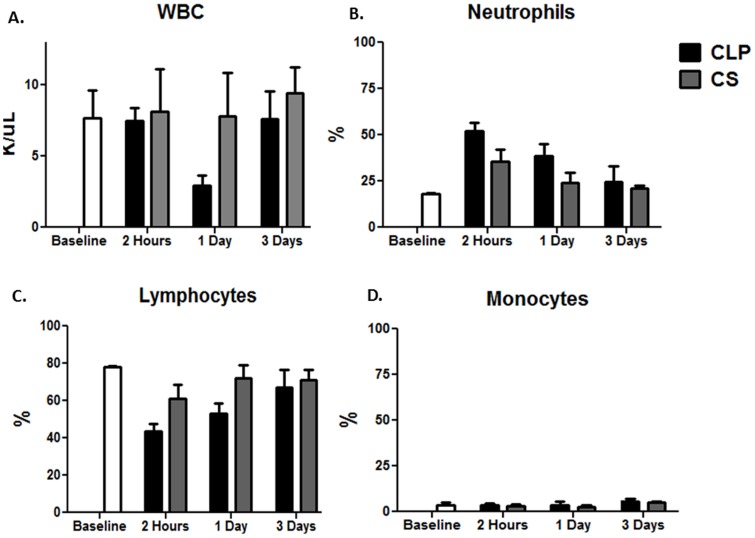
WBC count and leukocyte subset differentials after sepsis. Both the model and the time were significant in **A**. total WBC count (p<0.01-time, p<0.01-model, 2-Way ANOVA), **B**. neutrophil percentage (p<0.0001-time, p<0.0001-model, 2-Way ANOVA), and **C**. lymphocyte percentage (p<0.0001-time, p<0.001-model, 2-Way ANOVA), but only time had a significant effect on the **D**. monocyte percentage (p<0.001-time, 2-Way ANOVA).

Interestingly, when examining the total WBC count and the differentials in both models, we found that the CLP model had a greater acute neutrophilia immediately after sepsis and persisting to one day, compared to the CS model, despite having a significantly lower magnitude inflammatory response from both a genomic and cytokine storm perspective (Figure 7).

## Discussion

Animal models, and more specifically, murine models, remain the mainstay for invasive and interventional studies in sepsis and trauma [19], as their uniform genetic background, ease of handling, availability of investigative reagents and widespread use has made them the animal of choice. Much of what we know about the biology of the immunological response to sepsis was first established with murine models. After the failures of hundreds of clinical trials utilizing various drug therapies to treat sepsis that had been successful in both rodents and primates, the steadfast reliance on murine models for sepsis research is being reevaluated [20], [21]. Additionally, the validity of murine models of sepsis and trauma has recently been called into question after studies have highlighted the immunologic and transcriptomic differences that occur between the murine and human response to trauma and sepsis [9], [22]. We have argued that when used appropriately and validated against specific components of the human sepsis response, research performed using murine models remain valuable tools to clinical and translational studies [10]. With that said, the current report emphasizes the potential limitations of murine models and the need to validate their utility in individual circumstances. Therefore, we set forth to help delineate the genomic differences occurring between two models of murine sepsis: the most commonly used model and ‘gold-standard’, CLP, and a less commonly used model CS [11].

The CLP model of murine intra-abdominal sepsis, developed in the 1980s, is considered the gold-standard model of murine sepsis and consists of a combination of an ischemic tissue injury coupled with autologous polymicrobial infection [15]. CLP is considered a host-barrier disruption model and is thought to mimic a perforated viscous, endogenous fecal contamination and variable progression of disease, which occurs in humans [21]. Although the model is unique in that the researcher can control the magnitude of disease by varying the needle size (and thus the size of the insult), it has been demonstrated that surgical variability and technique can play a significant role in outcome as well [23]. CLP induces an immune response that is characterized by an early pro-inflammatory phase, as is evident by up regulation of various interleukins, TLRs and acute phase response pathways (Figure 5 and Table S1) leading to the migration of innate immune effector cells to sites of inflammation. There is a simultaneous anti-inflammatory response, including the production of anti-inflammatory cytokines IL-10 (Figure 5), as well as overexpression of genes involving the IL-10 signaling pathway (Figure 5) [15]. This is in concordance with the representation of a new SIRS-CARS model in which there is simultaneous activation of both inflammatory and anti-inflammatory pathways [7], [24], [25]. Criticisms of the CLP model involve the difficulty of controlling the magnitude of the septic challenge, in that even though the size of the needle can be varied, it is impossible to control the rate and amount of material released from the perforated viscous [26].

Through our studies of polymicrobial sepsis in neonatal mice, we have also utilized the cecal slurry (CS) model of intra-abdominal sepsis [14]. We have found this to be a reproducible model of intra-abdominal sepsis that could be utilized in mice as young as 5–7 days old with less risk of abandonment or cannibalism by the mother mouse that can occur after survival surgery. This model, originally utilized in pigs, consists of a fixed weight-based intraperitoneal injection of fecal contents suspended in dextrose, and was modified by our lab to be used as a murine model of sepsis [14], [27], [28]. This model uses the exogenous administration of fecal contents from a donor mouse, leading to a polymicrobial insult and has been thought to overcome the difficulties found in CLP in controlling the release of fecal material into the peritoneal cavity from the perforated viscous [26]. Similar to the CLP model, it induces an early pro-inflammatory phase; however, the degree of inflammation is greater than observed in CLP (Figure 6 and Figure 7), consistent with the initial recognition of more fecal contents.

Perfunctory examination of both the CS and CLP models would lead one to conclude that both are valid models of intra-abdominal sepsis, as both introduce peritoneal contamination with polymicrobial inoculum. Additionally, researchers typically consider both of these models to be similar in the fact that they produce a biphasic response to sepsis including an early, hyper-inflammatory phase, followed by prolonged illness with abscess formation [8], [27], [29]. More specifically, the main difference lies in the fact that the CLP model has peritonitis in the presence of devitalized tissue, which can be helpful if the researcher is studying similar human diseases such as diverticulitis or volvulus. Similar to the colon-ascenders stent peritonitis model (CASP), another model of peritonitis, the CS model has higher levels of inflammatory cytokines 24 hours after sepsis (Figure 6A). From a genomic pathway standpoint, both models tend to globally illicit early activation of innate inflammatory pathways and more pronounced suppression of adaptive immune suppression, including T and B cell response pathways, as is characteristic after human injury or sepsis [30]–[33]. Additionally, very early after sepsis, the CS model more readily exhibits the simultaneous expression of classical inflammatory and anti-inflammatory as well as some suppression of adaptive immune pathways, as was shown to occur after human injury in the Glue Grant [30].

In a recent human study by our group, we noted a dramatic similarity in the leukocyte transcriptome between patients with burn injury or trauma. The Pearson correlation coefficient for leukocyte genes whose expression changed was 0.91 (p<0.0001) [9]. In this study we demonstrated that the leukocyte genomic response to CLP and CS models of intra-abdominal sepsis in mice are much less similar, with genome wide Pearson correlations of only 0.54 (p<0.0001). Although it must be taken into consideration that the methodology of determining the genes relevant for these Pearson correlations was somewhat different, the results are still quite striking. Overall, it may suggest that the murine response to sepsis may be more variable than the human response to multiple different inflammatory events.

The differences in gene expression between CS and CLP suggest variable activation of specific immune pathways, many of which are unique to each model. The critical findings reported here are that the murine response to intra-abdominal sepsis can be manifested through different mechanisms at the level of the transcriptome depending on which sepsis model is utilized. These analyses reveal that the murine blood leukocyte response to sepsis is variable depending on which model of sepsis is employed. Overall both models tend to show an increase in transcription of genes involved in upregulating the innate immune response and a down regulation of genes regulation of the adaptive immune response, as has previously been shown to occur in human injury [30], although each to a different extent. The CS slurry model of sepsis, based on both plasma cytokine concentrations and the leukocyte transcriptome, appears to emphasize the early inflammatory component of sepsis, and the magnitude of the response is markedly higher one day after sepsis. In contrast, the CLP model of sepsis, which arises from a nidus of infection, appears to favor, at least at the level of the leukocyte transcriptome, the increased expression of immune suppressive pathways, such as IL-10 signaling; and early, short-lived activation of innate inflammatory pathways. For example, one day after sepsis, the CLP model has inhibition of the NFKB signaling pathway, whereas the CS model has continued activation of this pathway at one and three days (Figure 5 and Supplemental Figure 1). Additionally, the CLP model has more prominent expression of genes leading to the suppression of adaptive immune pathways such as those involved in apoptosis and Cd28 signaling, B-cell receptor and T-cell receptor signaling, as well as T helper cell and T lymphocyte pathways, especially one and three days after sepsis (Figure 5).

Many believe that the differences in the transcriptomic response to sepsis can be influenced by the absolute number of circulating WBCs or the differential make-up of the circulating leukocytes. When examining the total WBC count and the leukocyte subset differentials, we found that indeed both the model and the time had a significant effect (p<0.01, 2-Way ANOVA, Figure 7) on total WBC count (Figure 7A), neutrophil (Figure 7B), and lymphocyte percentage (Figure 7C), but only time had a significant effect on the monocyte percentage (Figure 7D). Although there were differences in the both the absolute number of WBCs as well as the percentages of circulating neutrophils and lymphocytes, but not monocytes, in the mice undergoing either CS or CLP after sepsis (Figure 7), we do not believe that these differences can fully account for the diverse transcriptomic responses as the magnitude of the transcriptomic changes are much greater than the differences in leukocyte patterns. Likewise, the CLP model has a greater increase in circulating neutrophils after sepsis; however, the CLP model has a decreased magnitude of the inflammatory cytokine and genomic response compared to CS (Figure 6, Figure 7). Additionally, in our previously published studies comparing the genomic response to sepsis or trauma between different groups, we have shown several times that the transcriptomic differences or similarities between the groups cannot be easily explained by differences or similarities in the prevalent type of circulating leukocyte after injury or the absolute WBC [34], [35]. For example, there are no significant differences in the circulating WBC differential percentages in neutrophils, lymphocytes, or monocytes between neonates and young adult or elderly mice, despite significant differences in their circulating WBC transcriptome and activated pathways [35].

The concluding message from these studies is that different models of murine polymicrobial sepsis are not interchangeable, and each model has its unique characteristics. Although recognized that both models do not replicate severe human sepsis in its entirety and vary among themselves, we would conclude that investigators will need to validate individual responses to severe sepsis in their murine model, especially when invasive or interventional studies are to be subsequently performed. Depending on the question being asked, each of these models offers unique and different targets for intervention.

## Supporting Information

Table S1 Immune and inflammatory related genes that are unique to the CLP model of intra-abdominal sepsis. Red signifies fold up regulation and blue signifies fold down regulation from control gene expression.
(DOC)

Table S2 Immune and inflammatory related genes that are unique to the CS model of intra-abdominal sepsis. Red signifies fold up regulation and blue signifies fold down regulation from control gene expression.
(DOCX)

Table S3 Immune and inflammatory related genes that are unique to the CS model of intra-abdominal sepsis. Red signifies fold up regulation and blue signifies fold down regulation from control gene expression.
(DOCX)
