# Supplementary material for: Host Responses to Sepsis Vary in Different Low-Lethality Murine Models
Source: PLoS One. 2014 May 1;9(5):e94404. doi: 10.1371/journal.pone.0094404 (PMC4006924; doi:10.1371/journal.pone.0094404)
Supplement: Table S1 — Immune and inflammatory related genes that are unique to the CLP model of intra-abdominal sepsis. Red signifies fold up regulation and blue signifies fold down regulation from control gene expression. (DOC) [file pone.0094404.s001.doc]

**Table S1**. Immune and inflammatory related genes that are unique to the CLP model of intra-abdominal sepsis. Red signifies fold up regulation and blue signifies fold down regulation from control gene expression.

| **Symbol** | **2 Hours** | **1 Day** | **3 Days** |
| --- | --- | --- | --- |
| **Bax** | **1.4** | **3.6** | **1.6** |
| **Casp12** | **-1.4** | **-1.9** | **-2.3** |
| **Casp14** | **-1.2** | **-1.7** | **-1.8** |
| **Ccr1** | **16.8** | **13.7** | **10.1** |
| **Ccr2** | **4.2** | **17.6** | **4.2** |
| **Ccr9** | **-2.1** | **-1.9** | **-3.4** |
| **Cebpb** | **12.4** | **11.2** | **5.9** |
| **Cxcl10** | **3.4** | **1.1** | **-1.3** |
| **Cxcl15** | **-1.1** | **1.6** | **1.6** |
| **H2-DMa** | **-3.6** | **-2.8** | **-3** |
| **H47** | **1.8** | **3.4** | **1.8** |
| **Hmgb2** | **12.3** | **16.1** | **16.2** |
| **Hp** | **7.5** | **10** | **4.6** |
| **Il10rb** | **4.2** | **5.8** | **3.7** |
| **Il15** | **2.6** | **3.4** | **4.1** |
| **Il17re** | **-1.1** | **-1.4** | **-1.4** |
| **Il18rap** | **3.5** | **3** | **2.6** |
| **Il1b** | **10.8** | **3.3** | **2.9** |
| **Il1r2** | **25.2** | **13.6** | **3.7** |
| **Il1rap** | **5.4** | **5.4** | **7.3** |
| **Tlr2** | **11.7** | **3.7** | **3.4** |
| **Tlr3** | **1.2** | **1.8** | **1.6** |
| **Trem1** | **12.5** | **6** | **4.6** |
