# Supplementary material for: Host Responses to Sepsis Vary in Different Low-Lethality Murine Models
Source: PLoS One. 2014 May 1;9(5):e94404. doi: 10.1371/journal.pone.0094404 (PMC4006924; doi:10.1371/journal.pone.0094404)
Supplement: Table S2 — Immune and inflammatory related genes that are unique to the CS model of intra-abdominal sepsis. Red signifies fold up regulation and blue signifies fold down regulation from control gene expression. (DOCX) [file pone.0094404.s002.docx]

**Table S2**. Immune and inflammatory related genes that are unique to the CS model of intra-abdominal sepsis. Red signifies fold up regulation and blue signifies fold down regulation from control gene expression.

| **Symbol** | **2 Hours** | **1 Days** | **3 Days** |
| --- | --- | --- | --- |
| **Arg1** | **-1.3** | **21.9** | **24** |
| **Bcl2** | **4.7** | **2.4** | **1.5** |
| **Cd33** | **3.3** | **3.7** | **4.5** |
| **Cd36** | **-8** | **-4.6** | **-4.6** |
| **Cd40** | **-5.5** | **-3.8** | **-4.5** |
| **Cebpd** | **-1** | **-1** | **3.4** |
| **Cxcl9** | **2.6** | **2.2** | **1.9** |
| **Cxcr3** | **-2.6** | **-3.3** | **-1.7** |
| **Cxcr6** | **-3.5** | **-3.4** | **-3.9** |
| **Cxcr7** | **2.2** | **16.8** | **11.2** |
| **H2-Ab1** | **-12.6** | **-7.9** | **-14.6** |
| **H2-Ab1** | **-25.6** | **-16.7** | **-19.7** |
| **H2-Oa** | **-2.4** | **-4.2** | **-4.5** |
| **H2-Ob** | **-5.2** | **-7** | **-5.9** |
| **H47** | **1.8** | **4.3** | **3** |
| **Hdac11** | **-1.1** | **-1.3** | **2.1** |
| **Hdac7** | **-1.2** | **-2.1** | **-1.6** |
| **Ifngr2** | **1.5** | **1** | **1.8** |
| **Il12a** | **-1.7** | **-1.5** | **-4.2** |
| **Il13ra1** | **-1.1** | **2** | **18.4** |
| **Il15ra** | **1.6** | **4.8** | **2** |
| **Il16** | **-4.7** | **-3.8** | **-6.5** |
| **Il17a** | **1.9** | **6** | **2.3** |
| **Il1rl1** | **4** | **2.7** | **1.6** |
| **Il1rl2** | **1.7** | **4.1** | **1.4** |
| **Il6st** | **2** | **1.9** | **-1.3** |
| **Ltf** | **16.8** | **30.7** | **15.2** |
| **Tlr7** | **2** | **4.2** | **1.8** |
