# Supplementary material for: Host Responses to Sepsis Vary in Different Low-Lethality Murine Models
Source: PLoS One. 2014 May 1;9(5):e94404. doi: 10.1371/journal.pone.0094404 (PMC4006924; doi:10.1371/journal.pone.0094404)
Supplement: Table S3 — Immune and inflammatory related genes that are unique to the CS model of intra-abdominal sepsis. Red signifies fold up regulation and blue signifies fold down regulation from control gene expression. (DOCX) [file pone.0094404.s003.docx]

**Table S3**. Immune and inflammatory related genes that are unique to the CS model of intra-abdominal sepsis. Red signifies fold up regulation and blue signifies fold down regulation from control gene expression.

| **Symbol** | **CS 2hr** | **CS 1D** | **CS 3D** | **CLP 2hr** | **CLP 1D** | **CLP 3D** |
| --- | --- | --- | --- | --- | --- | --- |
| **Bcl2l11** | **10.3** | **4.4** | **2.2** | **1.5** | **-1.8** | **-3.2** |
| **Ccl12** | **2.3** | **156.6** | **3.4** | **2.1** | **17.2** | **1.2** |
| **Ccl4** | **14** | **15.1** | **1.8** | **5.6** | **2.4** | **-1.9** |
| **Ccl6** | **-1.8** | **1.9** | **3.4** | **1.8** | **4.6** | **2.6** |
| **Ccl7** | **1.2** | **10.1** | **1.4** | **2.3** | **6.9** | **1.4** |
| **Ccr5** | **3.1** | **6.9** | **3.5** | **4.6** | **18.3** | **2.7** |
| **Ccr6** | **-2.2** | **-4.4** | **-3.7** | **-1.7** | **-4.2** | **-1.7** |
| **Ccrl2** | **4** | **2.3** | **-2.5** | **4.8** | **1.4** | **-3** |
| **Cd14** | **47.9** | **27.4** | **7.2** | **19.7** | **3** | **1.3** |
| **Cxcl2** | **313.6** | **360.8** | **21.6** | **78.8** | **19.3** | **1.7** |
| **Cxcl3** | **263.1** | **776.3** | **4.7** | **252.8** | **57.2** | **1.3** |
| **Cxcl5** | **-1.1** | **5.8** | **1.6** | **1.7** | **3.5** | **1.5** |
| **H2-DMb2** | **-6.4** | **-6.1** | **-20.1** | **-3.7** | **-8** | **-1.7** |
| **Il10** | **7.8** | **28.6** | **2.7** | **1.6** | **3.1** | **-1.3** |
| **Il16** | **-2.2** | **-2.9** | **-1.9** | **-1.6** | **-2.4** | **-1.3** |
| **Il18r1** | **2.3** | **1.1** | **1** | **-1.1** | **-2.5** | **-1.7** |
| **Il1a** | **7.2** | **9.5** | **2.1** | **3.3** | **1.1** | **-1.5** |
| **Il1rn** | **16.5** | **24.4** | **2.4** | **33.9** | **12.1** | **2.6** |
| **Il23a** | **1.3** | **-2.2** | **-1.5** | **1.3** | **-1.7** | **-1.7** |
| **Il6** | **31.1** | **173.8** | **7.1** | **14.8** | **3.3** | **2** |
| **Ilf3** | **-1.3** | **-1.4** | **-2.5** | **-1.4** | **-1.6** | **-1.4** |
| **Irak3** | **7.3** | **8.2** | **1.7** | **3.7** | **2** | **-2.5** |
| **Irg1** | **266.6** | **100.2** | **19.4** | **40.6** | **4.5** | **1.7** |
| **MIP-1α** | **20.2** | **31.3** | **-1.6** | **12.7** | **3.1** | **-3.8** |
| **Socs3** | **37.2** | **23.9** | **8.5** | **10.3** | **3.6** | **2.1** |
| **Tlr4** | **1.3** | **2.3** | **2.7** | **3.1** | **7.2** | **3.6** |
| **Tnf** | **3.1** | **2.7** | **-1.2** | **1.5** | **-1.2** | **-2.2** |
| **Traf1** | **8.3** | **3.8** | **-1.1** | **-1.1** | **-4.3** | **-3.7** |
| **Trem1** | **4.2** | **3** | **4.6** | **4.4** | **1.5** | **1** |
